# First case report of hospital staff infestation with cat flea (Ctenocephalides felis) in Iran

**DOI:** 10.3205/dgkh000432

**Published:** 2023-02-27

**Authors:** Saeid Amini Rarani, Mehdi Azami, Fatemeh Kiani, Tahereh Basir Kazeroni

**Affiliations:** 1 Nursing and Midwifery Care Research Center, Department of Operating Room, Isfahan University of Medical Sciences, Isfahan, Iran; 2 Skin Diseases and Leishmaniasis Research Center, Isfahan University of Medical Sciences, Isfahan, Iran; 3 Department of Medical Parasitology and Microbiology, Hojjatieh Medical Diagnostic Laboratory, Hojjatieh Hospital, Isfahan, Iran; 4 Basir Laboratory Research and Development Center, Basir Medical Diagnostic Laboratory, Isfahan, Iran

**Keywords:** cat flea, Ctenocephalides felis, health care worker infestation, Iran

## Abstract

**Background:**

Cat fleas (*Ctenocephalides felis*) are the most common ectoparasites of domestic cats and dogs worldwide. They can parasitize humans in many regions of the globe. Hospital infestation with fleas has not been reported in Iran, and the number of reported cases in the world is very low.

**Case presentation:**

Here we report and describe a hospital infestation with cat fleas in a number of health-care service personnel and nurses, which led to the development of skin lesions and severe itching.

**Conclusion:**

Diagnosing the parasite, removing it, and good health and medical management lead to satisfactory outcomes.

## Zusammenfassung

**Einleitung:**

Katzenflöhe (*Ctenocephalides felis*) sind weltweit die häufigsten Ektoparasiten von Hauskatzen und Hunden und können in vielen Regionen der Welt den Menschen parasitieren. Über einen nosokomialen Ausbruch mit Flöhen wurde im Iran noch nicht berichtet, und die Zahl der weltweit gemeldeten Fälle ist sehr gering.

**Präsentation der Fälle:**

Es wird über einen Befall mit Katzenflöhen beim Service- und Pflegepersonal berichtet, der zur Entwicklung von Hautläsionen und starkem Juckreiz führte.

**Schlussfolgerung:**

Die Diagnose des Parasiten, seine Bekämpfung und ein gutes medizinisches Management führten zu zufriedenstellenden Ergebnissen.

## Introduction

Fleas are small insects about 1–4 mm long. These wingless blood-sucking insects have an oval-shaped body that is compressed on the sides, and they also have long hind legs made for jumping. So far, approximately 2,574 species of fleas in 16 families and 238 genera have been reported in the world, and a small number of them exist near humans [1]. In general, three types of fleas have health and medical importance for humans: human fleas, dog and cat fleas, and rat fleas.

Fleas have a global distribution and are carriers of several important zoonosis, including plague and endemic typhus. These parasites play an important role in the transmission of various viral, bacterial, rickettsia and tapeworm agents in humans and animals [2]. 

The wide host range of fleas enables them to easily live with different hosts. In addition to the role of the flea in the transmission of some pathogenic agents, allergic dermatitis caused by its bite is also important. When fleas pierce the host with highly specialized parts of their mouths, flea allergic dermatitis occurs, which is caused by substances in flea saliva. Itching papules is one of the most painful symptoms of flea bites in the host [2]. Therefore, their control in medicine and veterinary medicine is important.

Based on the available information, cases of hospital contamination with fleas are very rare. Therefore, obtaining accurate information in this field can be of great help to health-care workers and facility managers in designing disease prevention strategies against the bites of these insects.

## Case Report

**Figure 1:**
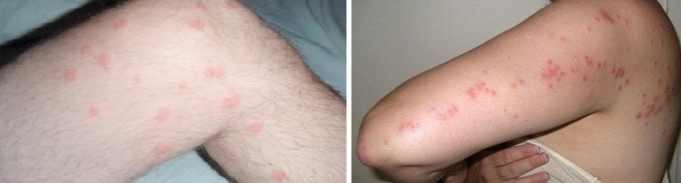
Urticaria-like lesions caused by flea bites on the knee, thigh, elbow and back of the patients

In December 2022, four members of the hospital service personnel were referred to the laboratory by the health unit of the hospital, due to severe itching that had lasted for more than two weeks. In further investigations, it was found that 2 nurses also had similar symptoms. All subjects reported severe itching, especially in the neck, abdomen, elbows, knees, and thighs (Figure 1 (Fig. 1)). All of them complained of severe itching for several days, which was worse during the day than at night. However, in some patients, there was no difference in the intensity of itching at night or during the day. Other family members of the patients were not affected. None of the patients had a pet dog or cat. Wide, pink lesions (diameter ca. 0.4 cm) similar to urticaria were observed in the elbow, neck, abdomen, knee and thigh areas of the afflicted. No lesions were observed in the genital areas of the subjects. None of the subjects had a history of skin or parasitic diseases. In the diagnosis of lesions, popular urticaria and popular eczema were considered.

Since all the examined individuals had similar lesions and were infected at the same time and place, a health inspection was conducted with the health officials from the workplace of these patients. An examination of the sweepings from the floor of the workplace of the patients revealed several insects which were small, black and less than 0.5 cm in size.

**Figure 2:**
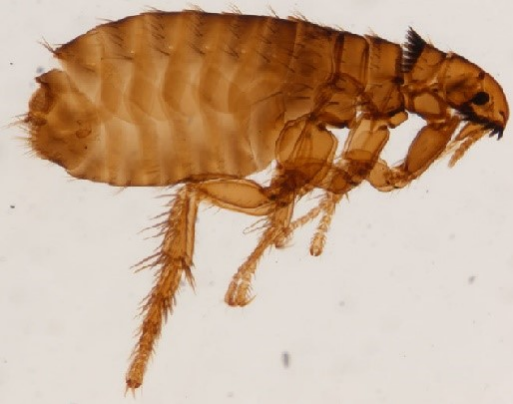
Cat flea (*Ctenocephalides felis*) isolated from dustpan contents swept from the floor of the hospital ward

The collected insects were examined by a parasitologist in the laboratory. The results of microscopic examinations found them to be reddish-brown wingless insects with numerous large spines on the head (genal comb) and thorax (pronotal comb). Other features were the short bristle on the dorsal margin of the hind tibia and the anteriorly pointing elongated frons. These morphological features confirmed identification as *Ctenocephalides felis* (Figure 2 (Fig. 2)), as previously described by Iyengar [3].

The infection control team informed the infected ward and all the patients and their equipment were transferred to the non-infected ward. Then, the floor, walls, furniture, and beds were sprayed with Clean-Up^TM^ II Pour-On Insecticide with IGR poison containing permethrin and diflubenzuron; spraying was repeated after two weeks.

## Discussion

Fleas are hematophagous ectoparasites on warm-blooded hosts and they are a matter of high importance both in the human and veterinary medical fields. Fleas are known as a vector of some important diseases, including plague, murine typhus, tularemia and dipylidiasis [4]. 

Every year, these insects cause irreparable damage to humans and animal industries. In addition, flea bites can lead to hypersensitivity responses, allergies and dermatitis. When fleas pierce the skin of the host with their highly specialized mouthparts, a phenomenon called Flea Allergy Dermatitis occurs originating from substances in flea saliva due to flea bite. The most common skin manifestation caused by flea bites is papular urticaria, which is an allergic reaction that leads to severe itching than can last for several weeks [5].

Studies reporting fleas as a nosocomial infestation are very few. In a Malaysian study, a number of hospitalized patients, nurses and health service personnel were infected with this parasite [6]. Similar to our study, all people infected with flea bites had papular urticaria lesions and severe itching for more than a month. In this study, after the detection of insects in hospital wards, spraying with methoprene and pyriproxyfen was performed and repeated two weeks later. 

The report of Yousefi et al. [7] describes a 28-year-old woman who was referred due to skin discomfort, itching and anxiety, especially at night, as well as an uncontrollable desire to scratch the skin lesions on the hips and back areas. In this investigation, the insect was isolated by the patient and sent to the laboratory, which confirmed the presence of cat fleas [7]. Another study reported about a 21-year-old woman with severe skin reactions on her right forearm. After initial examinations, the symptoms were recognized as a skin reaction caused by *Ctenocephalides felis* [8]. 

Noor Hayati et al. [9] reported macular papular rash caused by *Ctenocephalides felis* bites among students. Also, Chin et al. [10] showed the infestation of students with *Ctenocephalides felis* in Kuala Lumpur, Malaysia. 

Fular et al. [11] reported small, external papules caused by *Ctenocephalides felis* in a family in India. In that study, it was found that the person bitten by cat fleas was highly sensitivity to flea saliva, which led to severe skin blisters [11]. Although deaths due to flea infestations are rarely reported, Yeruham et al. [12] described severe anemia and finally death in a Palestinian youth. 

Kramer et al. [13] documented a patient with cat-flea infestation who was admitted to the university medical center in Greifswald, Germany. Through immediate diagnosis and the following measures, no other person was infested. The patient-side measures comprise:

- Isolation in single room;
- daily bath or shower including scalp hair, until flea infestation ends;
- parallel to bath or shower, underwear is washed and, if possible, outer clothing at ≥50°C, outer clothing is cleaned with a vacuum cleaner; in case of massive infestation, thermal disinfection or treatment with steam cleaner (≥65°C);
- if a patient has not yet been safely decontaminated but must leave the single room for medical reasons, s/he is dressed in a single-use overall;
- if flea infestation is only known after admission, check fellow patients for flea infestation before removing them from the patient's room and have them bathe or shower extensively, including head hair;
- visually inspect bedding during flea removal, crush visible fleas; continue decontamination bath or shower;
- daily change of bed linen, sleeping clothes and towels.

The measures for the staff comprise:

- Limit care to as few nurses as possible.
- Wear protective gowns and disposable gloves.
- Before leaving the room, as well as subsequently until the patient is safely decontaminated, check themselves for flea infestation.
- Change of work clothes at least daily.
- Shower extensively at the end of each shift.

The environmental measures are as follows:

- During the period of flea infestation until discharge, set up an electronic flea trap in the patient's room.
- Vcuum the patient's room every day, especially thoroughly around the bed and under it.
- Linen should be transported in a sealed laundry bag.
- If possible, do not use insecticides; otherwise, new occupancy is only possible after the insecticide has worn off.
- After discharge, clean all textiles, mattresses and floors (strong vacuum cleaner) as well as cupboards and interior of bedside table.

## Conclusions

Flea nosocomial infestation may be caused by the presence of animals in or around the hospital. Also, keeping dogs and cats at home or in the workplace can contribute to the transmission of infestation. Health control and periodic care of animals as well as the use of insecticides play an important role in preventing the transfer of these insects to hospitals.

## Notes

### Competing interests

The authors declare that they have no competing interests.

### Funding

This research received no specific grant from any funding agency in the public, commercial, or not-for-profit sectors.

### Patient’s consent

Consent forms were obtained from the patients.

### Acknowledgement 

We thank the patients who participated in the study. Without their consent, it would not have been possible to collect the data presented in this article.
